# Near-Infrared Spectroscopy-Derived Dynamic Cerebral Autoregulation in Experimental Human Endotoxemia—An Exploratory Study

**DOI:** 10.3389/fneur.2021.695705

**Published:** 2021-09-10

**Authors:** Nick Eleveld, Cornelia W. E. Hoedemaekers, C. Ruud van Kaam, Guus P. Leijte, Judith M. D. van den Brule, Peter Pickkers, Marcel J. H. Aries, Natasha M. Maurits, Jan Willem J. Elting

**Affiliations:** ^1^Department of Neurology, University Medical Center Groningen, University of Groningen, Groningen, Netherlands; ^2^Department of Intensive Care Medicine, Radboud University Medical Center, Radboud University, Nijmegen, Netherlands; ^3^Radboud Center for Infectious Diseases (RCI), Radboud University Medical Center, Radboud University, Nijmegen, Netherlands; ^4^Department of Intensive Care Medicine, School of Mental Health and NeuroSciences (MHeNS), University Medical Center Maastricht (MUMC+), Maastricht University, Maastricht, Netherlands

**Keywords:** near-infrared spectroscopy, human endotoxemia model, dynamic cerebral autoregulation, sepsis, cerebral perfusion

## Abstract

Cerebral perfusion may be altered in sepsis patients. However, there are conflicting findings on cerebral autoregulation (CA) in healthy participants undergoing the experimental endotoxemia protocol, a proxy for systemic inflammation in sepsis. In the current study, a newly developed near-infrared spectroscopy (NIRS)-based CA index is investigated in an endotoxemia study population, together with an index of focal cerebral oxygenation.

**Methods:** Continuous-wave NIRS data were obtained from 11 healthy participants receiving a continuous infusion of bacterial endotoxin for 3 h (ClinicalTrials.gov NCT02922673) under extensive physiological monitoring. Oxygenated–deoxygenated hemoglobin phase differences in the (very)low frequency (VLF/LF) bands and the Tissue Saturation Index (TSI) were calculated at baseline, during systemic inflammation, and at the end of the experiment 7 h after the initiation of endotoxin administration.

**Results:** The median (inter-quartile range) LF phase difference was 16.2° (3.0–52.6°) at baseline and decreased to 3.9° (2.0–8.8°) at systemic inflammation (*p* = 0.03). The LF phase difference increased from systemic inflammation to 27.6° (12.7–67.5°) at the end of the experiment (*p* = 0.005). No significant changes in VLF phase difference were observed. The TSI (mean ± SD) increased from 63.7 ± 3.4% at baseline to 66.5 ± 2.8% during systemic inflammation (*p* = 0.03) and remained higher at the end of the experiment (67.1 ± 4.2%, *p* = 0.04). Further analysis did not reveal a major influence of changes in several covariates such as blood pressure, heart rate, PaCO_2_, and temperature, although some degree of interaction could not be excluded.

**Discussion:** A reversible decrease in NIRS-derived cerebral autoregulation phase difference was seen after endotoxin infusion, with a small, sustained increase in TSI. These findings suggest that endotoxin administration in healthy participants reversibly impairs CA, accompanied by sustained microvascular vasodilation.

## Introduction

Sepsis is defined as life-threatening organ dysfunction caused by a dysregulated host response to infection (1). Apart from systemic effects, sepsis patients may experience cerebral complications. Sepsis-associated encephalopathy (SAE) is a severe complication in sepsis (2) and is associated with increased mortality and long-term cognitive impairment. The pathophysiology of SAE is multifactorial, and inflammatory cytokines, alterations in cerebral metabolism, and blood–brain-barrier compromise have been described as etiological factors for the development of SAE (2). In addition, there is increasing evidence for alteration of the cerebral (micro-)circulation and cerebral autoregulation (CA) during sepsis, which may play an important role in SAE pathogenesis (3, 4).

The experimental human endotoxemia model is a standardized reproducible model in which healthy volunteers are challenged with endotoxin (lipopolysaccharide, LPS) to induce a systemic inflammatory response. This model can be used to study systemic inflammation and has been used to investigate several phenomena associated with sepsis, including cerebral perfusion, cerebral (micro-) vascular function, and SAE (5–9). In general, although alterations in CA are seen in patients with SAE, there are conflicting findings regarding CA in healthy participants undergoing experimental endotoxemia. Three studies have estimated the dynamic cerebral autoregulation (DCA) with transfer function analysis (TFA), in which the temporal relation was determined between oscillations in arterial blood pressure (ABP) and cerebral blood flow velocity (CBFv) measured with transcranial Doppler (TCD). DCA is quantified with “gain” and “phase difference”. Two earlier studies reported an improvement in DCA (reduced gain and increased phase difference) upon infusion of 2 ng/kg bacterial LPS (7, 10). In a recent study, Van den Brule et al. investigated DCA in an endotoxemia study with a continuous infusion of, in total, 4 ng/kg bacterial LPS. No changes in gain or phase difference were found (5).

CA has also been assessed with measurements of ABP and near-infrared spectroscopy (NIRS), a non-invasive technique that uses near-infrared light to calculate the concentration of hemoglobin (Hb) in the cerebral cortex (11, 12). Recently, a new DCA measurement model that employs only high-frequency continuous-wave NIRS measurements on the frontotemporal scalp was developed (13). In short, the temporal relation between the concentration of oxygenated (OxyHb) and deoxygenated hemoglobin (HHb) is determined with TFA, which results in phase difference values analogous to TFA for ABP and CBFv. Correction for transit time and blood flow/blood volume (TT-BF/BV) oscillations for phase difference values is needed, after which NIRS-derived DCA phase difference values were similar to the phase difference values obtained *via* the classic TCD- and ABP-based method in healthy volunteers during rest and hypercapnia (13).

The NIRS technique to assess DCA has several advantages over the technique using TCD and ABP: it requires little operator experience, is more comfortable for the participant, and allows for long-term measurements. These advantages could provide a higher signal-to-noise ratio for the NIRS compared to the TCD/ABP method. Further studies in clinically relevant populations are therefore warranted. In the current study, unilateral, frontotemporal NIRS measurements obtained from the same healthy participants that were studied by Van den Brule et al. during experimental endotoxemia (5) were analyzed to explore the temporal dynamics of NIRS-derived CA. We hypothesized that possible changes in CA can be detected more easily with NIRS than with TCD and ABP due to the aforementioned advantages of the NIRS-CA technique.

We compared the temporal dynamics of NIRS-derived dynamic cerebral autoregulation as characterized by TFA phase difference before, during, and after administration of endotoxin. Furthermore, we investigated the dynamics of the Tissue Saturation Index (TSI), a more common and clinically used NIRS measure of cerebral oxygenation, as the TSI is thought to be susceptible to changes in cerebral blood volume (CBV) or cerebral blood flow (CBF).

Lastly, the relation was explored between phase difference and TSI, and several potential confounders that have been described to influence cerebral perfusion and DCA: mean arterial pressure (MAP), PaCO_2_, pH, temperature, heart rate, inflammatory cytokine concentrations, and power in ABP and NIRS oscillations (14, 15).

## Materials and Methods

### Participants and Study Protocol

NIRS data from 11 participants were used from an existing dataset of healthy adult males undergoing an experimental human endotoxemia study [ClinicalTrials.gov NCT02922673 (16)]. Of the original 13 datasets, two participants were excluded because of the insufficient quality of NIRS data resulting from excessive movement artifacts.

**Figure 1:**
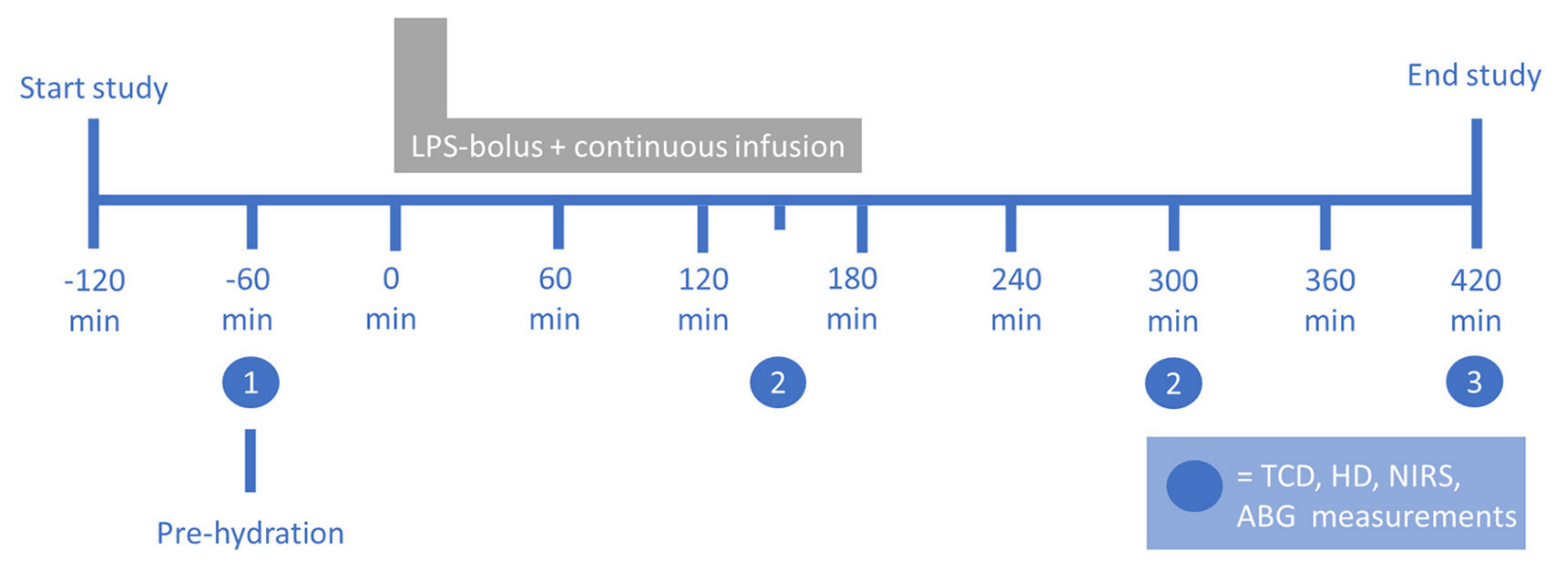
Setup of the human endotoxemia trial. The numbers indicate the measurements at (1) baseline, (2) systemic inflammation, and (3) the end of the experiment. ABG, arterial blood gas; HD, hemodynamic; LPS, lipopolysaccharide; NIRS, near-infrared spectroscopy; TCD, transcranial Doppler. Figure reproduced with permission from Figure 1 in Van den Brule et al., *Shock* (2018), doi: 10.1097/SHK.0000000000001003.

A comprehensive study protocol for the study has been published previously (5, 16). A summary of the study protocol is provided here. A continuous infusion of bacterial LPS was employed under extensive vital sign monitoring, with additional NIRS measurements. A schematic description of the protocol is provided in Figure 1. Data on age, body mass index (BMI), resting heart rate, MAP, and (tympanic) body temperature were obtained at baseline (*T* = −60 min). All participants received pre-hydration with 1.5 L of 2.5% glucose/0.45% saline solution in the hour before LPS administration, followed by 150-ml/h continuous hydration until the end of the experiment (5). Purified LPS (lipopolysaccharide, *E. coli Type O113*, lot no. 94332B4; List Biological Laboratories, Campbell, USA) was dissolved in normal saline (0.9%). An intravenous loading bolus of 1 ng/kg body weight was administered, followed by a continuous infusion of 1 ng/kg body weight/hour for a period of 3 h (5).

### Monitoring and Data Collection

Monitoring of clinical parameters included continuous invasive ABP, electrocardiography, arterial oxygen saturation (SpO_2_), intermittent tympanic temperature, TCD-derived cerebral blood flow velocity in the middle cerebral artery (MCA), arterial blood gas, and inflammatory cytokine concentrations (5, 16).

NIRS recordings were performed at four time points: 60 min before LPS administration (baseline), during systemic inflammation at 150 and 300 min after the initiation of LPS administration, and at the end of the experiment, 420 min after the initiation of LPS administration. A continuous-wave NIRS device (Portalite, Artinis Medical Systems, Elst, The Netherlands) was placed on the frontotemporal forehead. Near-infrared light was transmitted with wavelengths of 760 and 850 nm by a light-emitting diode and was received by a photodiode at 35-mm inter-optode distance. Data were obtained at 50-Hz sampling frequency with device-specific acquisition software (Oxysoft, Artinis Medical Systems, Elst, The Netherlands). Changes in OxyHb and HHb were derived using the modified Beer–Lambert Law (17).

All OxyHb and HHb data were systematically inspected for artifacts and noise with in-house developed Labview data visualization and correction software (Labview 2015, National Instruments, Austin, TX, United States), which has been used before by our group (13). The artifacts were visually identified and removed by linear interpolation as recommended by the international Cerebral Autoregulation Research Network (CARNet) (18). For longer artifacts (>5 s), artifact containing data were removed. A second reviewer visually inspected the resulting data for any remaining artifacts. At least 5 min of high-quality data were required for each time point as is recommended (18).

The mean cerebral TSI was calculated from the artifact-corrected OxyHb and HHb data using spatially resolved spectroscopy in Oxysoft, following the method of Matcher et al. (19, 20).

### Non-invasive, NIRS-Only-Derived Dynamic Cerebral Autoregulation

The relationship between OxyHb and HHb was determined with TFA following CARNet recommendations (18). Power spectral density and cross-spectral density estimates were determined using Welch's method (100-s epochs, 50% window overlap). Coherence, gain, and phase difference were computed and trichotomized into one of three frequency bands: very low frequency (VLF): 0.02–0.07 Hz, low frequency (LF): 0.07–0.2 Hz, and high frequency (HF): 0.2–0.5 Hz. Gain and phase difference values were excluded if the corresponding coherence values were below the window-dependent coherence threshold as defined in the CARNet White Paper (18).

The capillary TT-BF/BV correction method developed by our group was applied to the phase difference values after TFA calculation (13). In short, a linear trend line was fitted to the HF band phase difference values. Only data sections that yielded at least five consecutive frequency bins with significant coherence were used for line fitting, thereby avoiding inclusion of bins with significant coherence that arose by chance. Subsequently, this trend line was subtracted from the LF/VLF phase difference values, thereby obtaining the NIRS-derived TT-BF/BV-corrected CA estimation. NIRS-derived CA estimates were defined as TT-BF/BV-corrected VLF and LF phase differences. These were calculated for each time point for which NIRS data were available.

### Data Pooling

For the measurements at 150 min after the initiation of endotoxin administration, movement-related artifacts were prominent in some of the NIRS data (healthy volunteers getting sicker), so for this time point, data from only *n* = 7 participants were available. Although there was a difference in inflammatory response as measured by plasma cytokine concentration between 150 and 300 min after the initiation of endotoxin administration, inflammation at both time points was markedly higher than at baseline or at the end of the experiment as was reported previously (5). We therefore decided to pool the data at these two time points by averaging to obtain one “intra-inflammatory” measurement for each participant. If measurements were only available at either 150 or 300 min, these data were used. For further analysis, data were therefore obtained from three time points: (1) baseline (−60 min), (2) systemic inflammation, and (3) at the end of the experiment (420 min).

### Statistical Analysis

Data distribution was assessed for normality using histograms and the Shapiro–Wilk *W*-test and for sphericity using Mauchly's test. For normally distributed data, the between-time point differences were determined using a repeated-measures analysis of variance (rm-ANOVA). For non-normally distributed data, the non-parametric Friedman test was used. Paired *t*-tests and Wilcoxon signed rank tests were used for *post-hoc* testing in case of normally and non-normally distributed data, respectively. The significance level was set at α = 0.05. Pearson's correlation coefficient with Benjamini–Hochberg correction was used to explore the relation between the change in phase difference and TSI between different time points and changes in hemodynamic confounders related to cerebral perfusion and CA. Statistical analyses were performed using Stata/SE 16 (StataCorp., College Station, TX) and MATLAB 2019b (The MathWorks, Inc., Natick, MA).

## Results

### Baseline Characteristics and Hemodynamic Parameters

**Figure 2:**
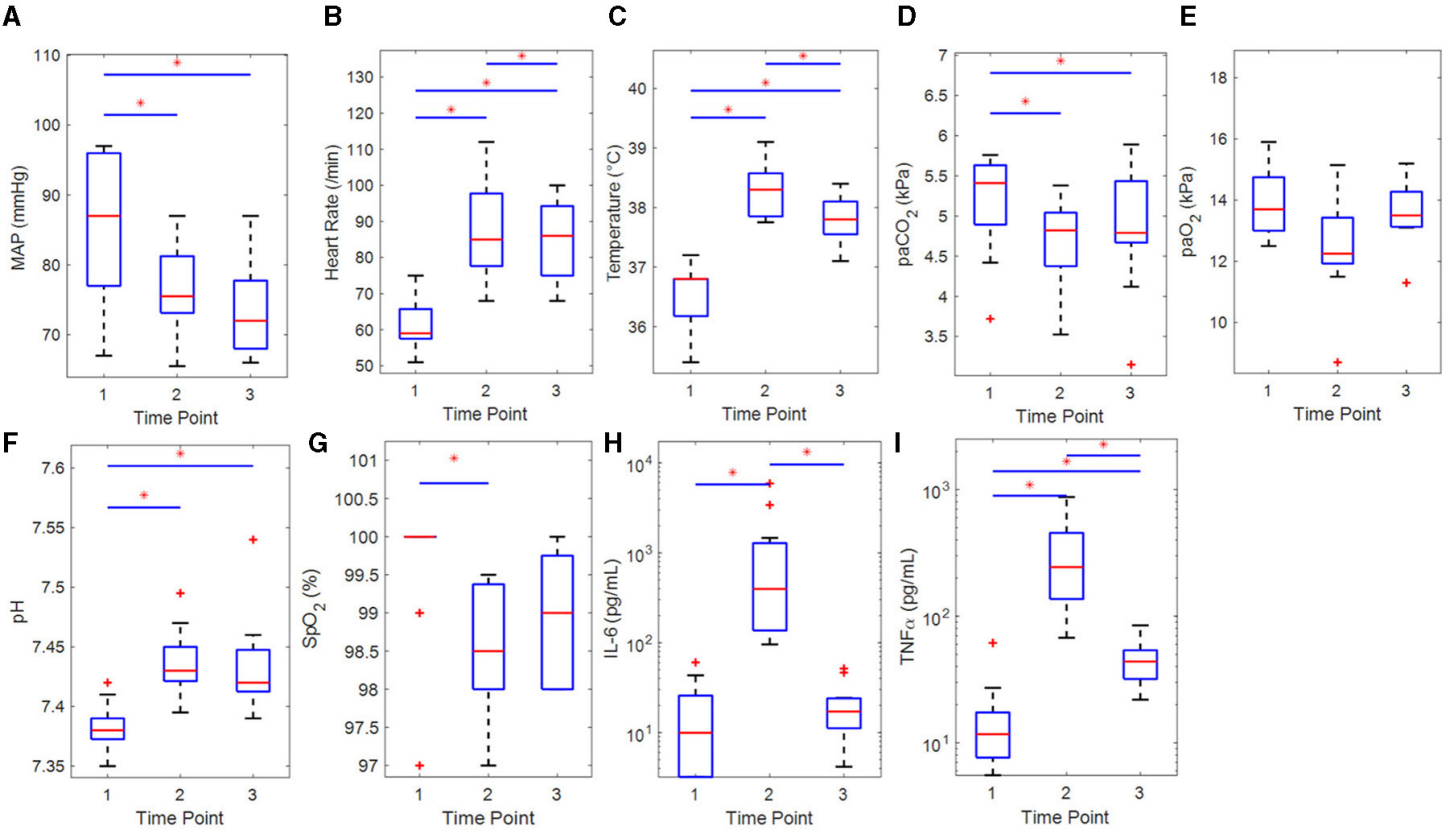
Boxplots of hemodynamic and clinical parameters of the participants (*n* = 11) for each time point with results of the statistical comparisons. **(A)** Mean arterial pressure (rm-ANOVA), **(B)** heart rate (rm-ANOVA), **(C)** temperature (Sign. Rnk), **(D)** PaCO2 (Sign. Rnk), **(E)** PaO2 (Sign. Rnk), **(F)** pH (Sign. Rnk), **(G)** SpO2 (Sign. Rnk), **(H)** IL-6 (Sign. Rnk), and **(I)** TNFα (Sign. Rnk). IL, interleukin; MAP, mean arterial pressure; PaO_2_/PaCO_2_, arterial partial pressure of oxygen/carbon dioxide; rm-ANOVA, repeated-measures analysis of variance; Sign. Rnk, Wilcoxon's signed rank test; SpO_2_, arterial saturation of oxygen; TNFα, tumor necrosis factor alpha. *p < 0.05 for difference between time points.

The participants were healthy young adults (22.4 ± 1.6 years old) with a BMI of 24.2 ± 2.4 kg/m^2^ and normal baseline physiological measurements as can be seen in Table 1. The hemodynamic parameters at each time point are provided in Table 1 and Figure 2. A sustained decrease in MAP and a reversible decrease in PaCO_2_ was observed, respectively. Heart rate, temperature, and pH showed a reversible increase.

**Table 1:** Hemodynamic and clinical parameters of the participants (*n* = 11) for each time point.

		**Time point 1 baseline**	**Time point 2 systemic inflammation**	**Time point 3 end of experiment**	***p*-value TP1 vs. TP2**	***p*-value TP1 vs. TP3**	***p*-value TP2 vs. TP3**
MAP (mmHg)	Mean ± SD	86.1 ± 10.7	76.6 ± 6.6	73.3 ± 6.5	0.0103	0.0032	0.2168
Heart rate (/min)	Mean ± SD	61.5 ± 6.9	88.7 ± 13.5	84.1 ± 10.7	0.0000	0.000	0.0427
Temperature (°C)	Median (IQR)	36.8 (36–36.8)	38.3 (37.9–38.6)	37.8 (37.5–38.1)	0.0010	0.0010	0.0186
SpO_2_ (%)	Median (IQR)	100 (100)	98.5 (98.0–99.5)	99 (98 - 100)	0.0020	0.0547	0.2891
pH	Median (IQR)	7.38 (7.37–7.39)	7.43 (7.42–7.45)	7.42 (7.41–7.45)	0.0010	0.0020	0.6563
PaO_2_ (kPa)	Mean ± SD	14.0 ± 1.2	12.5 ± 1.7	13.7 ± 1.0	N.S.	N.S.	N.S.
PaCO_2_ (kPa)	Median (IQR)	5.41 (4.73–5.69)	4.85 (4.36–5.09)	4.79 (4.65–5.47)	0.0020	0.042	0.4131
IL-6 (ng/ml)	Median (IQR)	9.9 (3.2–30.4)	395 (134–1467)	17.2 (10.3–24.2)	0.0010	0.4131	0.0010
TNFα (pg/ml)	Median (IQR)	11.7 (7.3–17.7)	243 (131–476)	43.8 (31.6–54.4)	0.0010	0.0029	0.0010

*IL, interleukin; IQR, inter-quartile range; MAP, mean arterial pressure; N.S., repeated-measures test statistic not significant; PaO_2_/PaCO_2_, arterial partial pressure of oxygen/carbon dioxide; p-value, post-hoc test p-value for difference between time points; SpO_2_, arterial saturation of oxygen; SD, population standard deviation; TNFα, tumor necrosis factor alpha; TP, time point*.

### NIRS-Derived Cerebral Autoregulation

**Figure 3:**
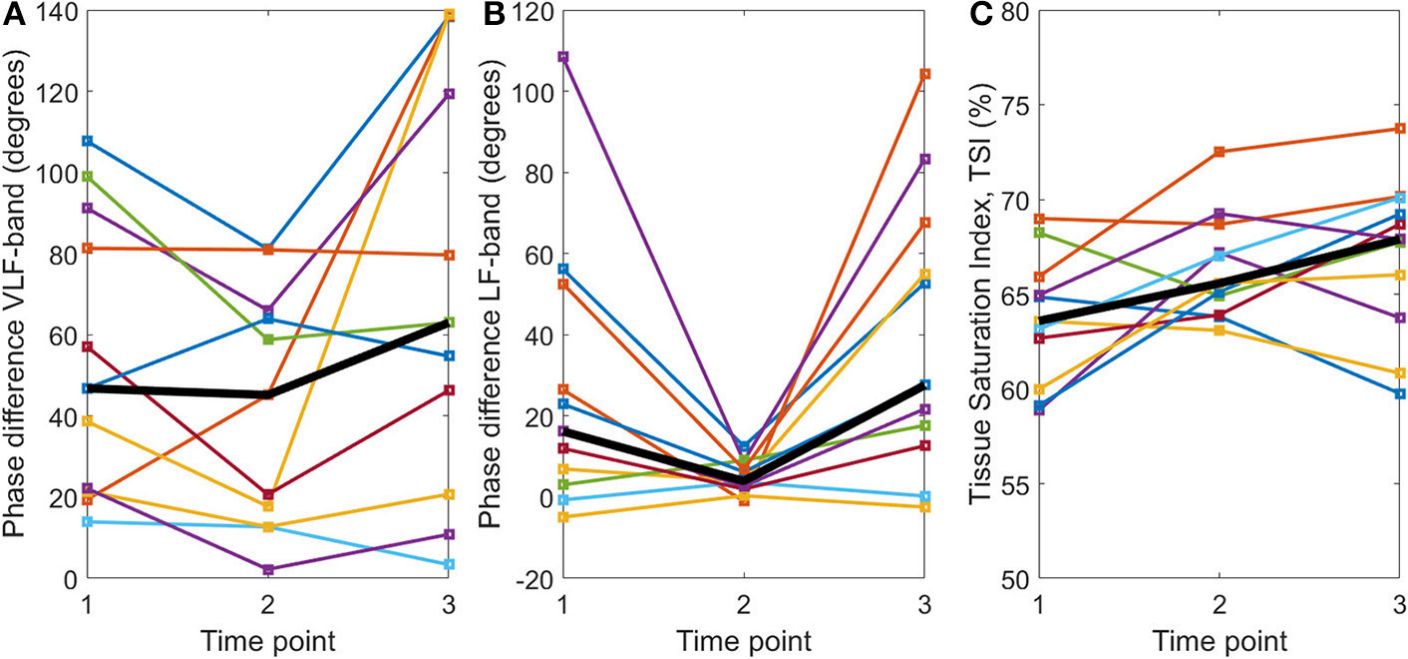
TT-BF/BV-corrected phase differences (degrees) between OxyHb and HHb for the **(A)** very low frequency and **(B)** low frequency bands and **(C)** mean tissue saturation index (%) calculated with spatially resolved spectroscopy at three time points: (1) baseline, (2) systemic inflammation, and (3) at the end of the experiment. The per time point group median is shown by the black line.

The NIRS measurement duration was 20.9 ± 7.4 min (mean ± SD). After the application of TT-BF/BV correction, phase difference values were obtained in the VLF and LF bands (data on coherence, gain, and phase difference for all frequency bands can be found in the Supplementary Material). Phase difference values are provided for each participant at each time point for both the VLF and LF frequency bands in Figures 3A,B, respectively. No statistically significant differences in VLF phase difference values were found between the different time points (χ^2^ = 5.10, *p* = 0.08, Friedman test), although a trend toward lower values at time point 2 could be observed. For the LF band, a statistically significant difference in phase difference values between the time points was found (χ^2^ = 7.82, *p* = 0.02). Median (IQR) LF phase difference values decreased from time point 1 (16.2°, 3.0–52.6°) to time point 2 (3.9°, 2.0–8.8°; *Z* = 2.13, *p* = 0.03). The LF phase difference values were also higher at time point 3 (27.6°, 12.7–67.5°) than at time point 2 (*Z* = −2.67, *p* = 0.005, all Wilcoxon signed rank tests). No significant difference between time points 1 and 3 was found (*Z* = −1.78, *p* = 0.08).

No correlation was found between changes in LF phase difference between the time points and changes in the following variables: LF band power spectral density (PSD) in OxyHb, HHb, or ABP or PaCO_2_, pH, and IL-6 or TNFα concentrations (all *R*^2^ <0.25, *p* > 0.1; see Supplementary Material for details). A positive correlation was found between changes in LF phase difference between time points 1 and 2 and changes in MAP (*R*^2^ = 0.50, *p* = 0.015). A negative correlation was found between changes in LF phase difference between time points 2 and 3 and changes in heart rate (*R*^2^ = 0.45, *p* = 0.024) and temperature (*R*^2^ = 0.65, *p* = 0.003). Only the latter correlation remained statistically significant after correcting for multiple comparisons.

### Tissue Saturation Index

TSI values are provided for each participant at each time point in Figure 3C. There was a significant difference in TSI between time points (*F* = 4.78, *p* = 0.02, rm-ANOVA). *Post-hoc* testing indicated that the TSI was higher at time points 2 (66.5 ± 2.8%) and 3 (67.1 ± 4.2%) than at time point 1 (63.7 ± 3.4%) [*t*_(10)_ = −2.46, *p* = 0.03 and *t*_(10)_ = −2.40, *p* = 0.04, respectively]. No significant differences between time points 2 and 3 were found [*t*_(10)_ = −0.68, *p* = 0.51].

No correlation was found between changes in TSI between the time points and changes in the following variables: LF band PSD in OxyHb, HHb, or ABP, PaCO_2_, temperature, heart rate, and IL-6 or TNFα concentrations (all *R*^2^ <0.25, *p* > 0.1) (Supplementary Material). A weak, negative correlation was found between changes in TSI between time points 1 and 2 and pH (*R*^2^ = 0.40, *p* = 0.04).

## Discussion

### Interpretation of the Results

In this study, NIRS-derived DCA estimations were obtained during experimental human endotoxemia with a continuous infusion of bacterial LPS. The TT-BF/BV-corrected phase difference values in the LF band were significantly lower during systemic inflammation compared with baseline and at the end of the experiment. In addition, the TSI increased significantly during systemic inflammation compared with baseline and remained significantly higher at the end of the experiment.

We found a reversible decrease in LF phase difference measured with NIRS, which suggests impaired CA during endotoxemia in healthy volunteers. The extensive physiological changes resulting from endotoxin administration could theoretically underlie the observed NIRS changes, but after extensive analysis, we found no unambiguous explanation for the observed decrease in LF phase difference measured with NIRS.

A decrease in LF phase difference between time points 1 (baseline) and 2 (systemic inflammation) correlated with a decrease in MAP, although this was not significant after correcting for multiple comparisons. Furthermore, although the LF phase difference increased between time points 2 and 3 (end of the experiment), MAP did not, attenuating the interpretation of a major influence of MAP changes on LF phase. Furthermore, an increase in LF phase correlated with a decrease in heart rate and temperature, although only the latter remained statistically significant after correcting for multiple comparisons and only between time points 2 and 3. There was no correlation with temperature and heart rate changes between time points 1 and 2, supporting the interpretation that these changes may not be an important confounder. Other potential confounders that we analyzed were changes in inflammatory cytokines (IL-6 and TNFα) and PaCO_2_. All these variables did not correlate with the changes in LF phase. Note that the absence of correlations with potential confounders in our small sample should not be interpreted as proof of the absence of any such relationship: it could be that there actually is a (weak) relationship that could be identified in larger samples.

Previous data by Brassard and colleagues support the idea that the improvement in dynamic CA that they found during experimental endotoxemia correlated with a decrease in carbon dioxide tension (7). The relation between hypocapnia and DCA phase difference is well recognized (21, 22). In our study, relative hypocapnia and alkalosis were also present, which are expected to induce an increase in LF band DCA phase difference. The opposite was observed (decrease in LF band phase). It could be that the effect of hypocapnia is masked in part by other physiological changes that occurred in our endotoxemia population, among others the decrease in MAP or a higher inflammatory response. Indeed the population studied by Brassard et al. had no decrease in MAP upon a lower bolus administration of 2 ng/kg of endotoxin.

The small but sustained increase in TSI that was observed may have resulted from microvascular vasodilatation, with concomitant increases in CBV and/or CBF. We could not further clarify this phenomenon based on our data, as we found only a weak, non-significant relation between the increase in TSI between time points 1 and 2 and an increase in pH. However, both an increase in TSI and a decrease in LF phase difference measured with NIRS are consistent with the interpretation of cerebral microvascular vasodilatation in response to endotoxin infusion.

No significant changes in VLF phase difference were found. We hypothesize that this is due to the limited reproducibility of VLF phase difference values using TFA (15, 23). A variation in LF phase difference values was seen between the participants showing an evident reversible decrease during endotoxin-induced systemic inflammation and the participants with consistently low phase difference values with limited variation. We hypothesize that CA was consistently “off” in the latter group, given the known non-stationarity of CA function, also in healthy participants (14).

### Comparison With TCD- and ABP-Derived DCA

The reversible decrease in NIRS-derived LF phase difference observed in this study has not been found with TFA using TCD and ABP measurements as reported previously (5). It could be that the autoregulatory dysfunction observed upon endotoxin administration mainly demonstrates itself in a patchy distribution in the cortical microvasculature, the effects of which cannot be easily found in TCD-based CBF velocity in the MCA. Another possibility is that some of the CA effects of endotoxin administration occur at the level of pre-capillary sphincters and not only at the level of the cerebral arterioles themselves (13, 24, 25). The NIRS measurements could be sensitive to both microvascular regulators. The OxyHb and HHb components are present proximal and distal to both regulators, making NIRS suitable for the detection of alterations in the function of both the arterioles and the pre-capillary sphincters. The TCD-derived CBFv could be only sensitive to the upstream resistance effects of the arterioles. Another explanation may be methodological. The variation in TFA analysis has been shown to decrease with measurement duration (23). In our study, the mean ± SD duration of the NIRS measurements was 20.9 ± 7.4 min, compared with 5 min as previously reported for TCD and ABP (5).

### Limitations

We recognize several limitations of our study. The sample size of 11 participants prevents an extensive, multivariate analysis of the data, and it may limit the external validity of the results. Furthermore, we pooled the NIRS parameters from two measurements (*T* = 150 and 300 min) due to movement artifacts in some measurements. This allowed an “intra-inflammatory” measurement for each participant, and it limited the number of statistical comparisons. We consider this justified and advisable, as the concentration of pro-inflammatory cytokines was high at both time points as published previously, indicating the systemic inflammatory response (5). Longer (>30 min) measurements would allow for more artifact-free data, but this was unfeasible in the current endotoxemia setup. The cerebral origin of the NIRS-only DCA measurements has previously been confirmed by comparison against TCD/ABP-derived DCA during rest and hypercapnia. However, we cannot exclude the confounding effects of changes in extracerebral perfusion that may have resulted from systemic microvascular vasodilation in the febrile phase of systemic inflammation in the current study. It should be mentioned that one participant was in a consistent hyperventilation (hypocapnic) state during the entire experiment (3.15 < PaCO_2_ <3.72 kPa) but was not excluded from the analysis to prevent selection bias. Lastly, the NIRS measurements are sensitive to movement artifacts, which was exemplified in our study as we had to exclude measurements from two participants. Movement-related artifacts may however be less pronounced in clinical target populations, including sedated patients in the ICU.

### Future Perspective

The NIRS-only technique is a non-invasive, easy-to-use modality for the measurement and monitoring of CA. This study provides first insights that NIRS-only-derived DCA changes during experimental human endotoxemia. It provides indications for an influence of bacterial endotoxin on DCA, which warrants further investigation in larger endotoxemia studies. Moreover, it could be interesting to investigate NIRS-derived DCA in a sepsis population, as the systemic inflammatory response in sepsis is higher, longer, and has a stronger hemodynamic effect than during experimental endotoxemia, and there is more variability in (cerebrovascular) outcome.

## Conclusion

Our findings suggest that endotoxin administration in healthy volunteers results in changes in cerebral microvascular autoregulation and microvascular vasodilation.

## Data Availability Statement

The raw data supporting the conclusions of this article will be made available by the authors, without undue reservation.

## Ethics Statement

The studies involving human participants were reviewed and approved by Commissie Mensgebonden Onderwoek regio Arnhem—Nijmegen. The patients/participants provided their written informed consent to participate in this study.

## Author Contributions

CH, PP, GL, and JB contributed to conception and design of the study. GL, JB, and CH performed the measurements. JE, NE, and NM performed the data-analysis and statistical analysis. NE drafted the manuscript. CH, GL, CvK, JvB, MA, JE, NE, and NM wrote sections of the manuscript. All authors contributed to the manuscript revision, read, and approved the submitted version.

## Funding

Graduate School of Medical Sciences, University of Groningen: Research grant NE.

## Conflict of Interest

The authors declare that the research was conducted in the absence of any commercial or financial relationships that could be construed as a potential conflict of interest.

## Publisher's Note

All claims expressed in this article are solely those of the authors and do not necessarily represent those of their affiliated organizations, or those of the publisher, the editors and the reviewers. Any product that may be evaluated in this article, or claim that may be made by its manufacturer, is not guaranteed or endorsed by the publisher.
