# Supplementary material for: Near-Infrared Spectroscopy-Derived Dynamic Cerebral Autoregulation in Experimental Human Endotoxemia—An Exploratory Study
Source: Front Neurol. 2021 Sep 10;12:695705. doi: 10.3389/fneur.2021.695705 (PMC8461327; doi:10.3389/fneur.2021.695705)
Supplement: Supplementary file 1 [file Data_Sheet_1.docx]

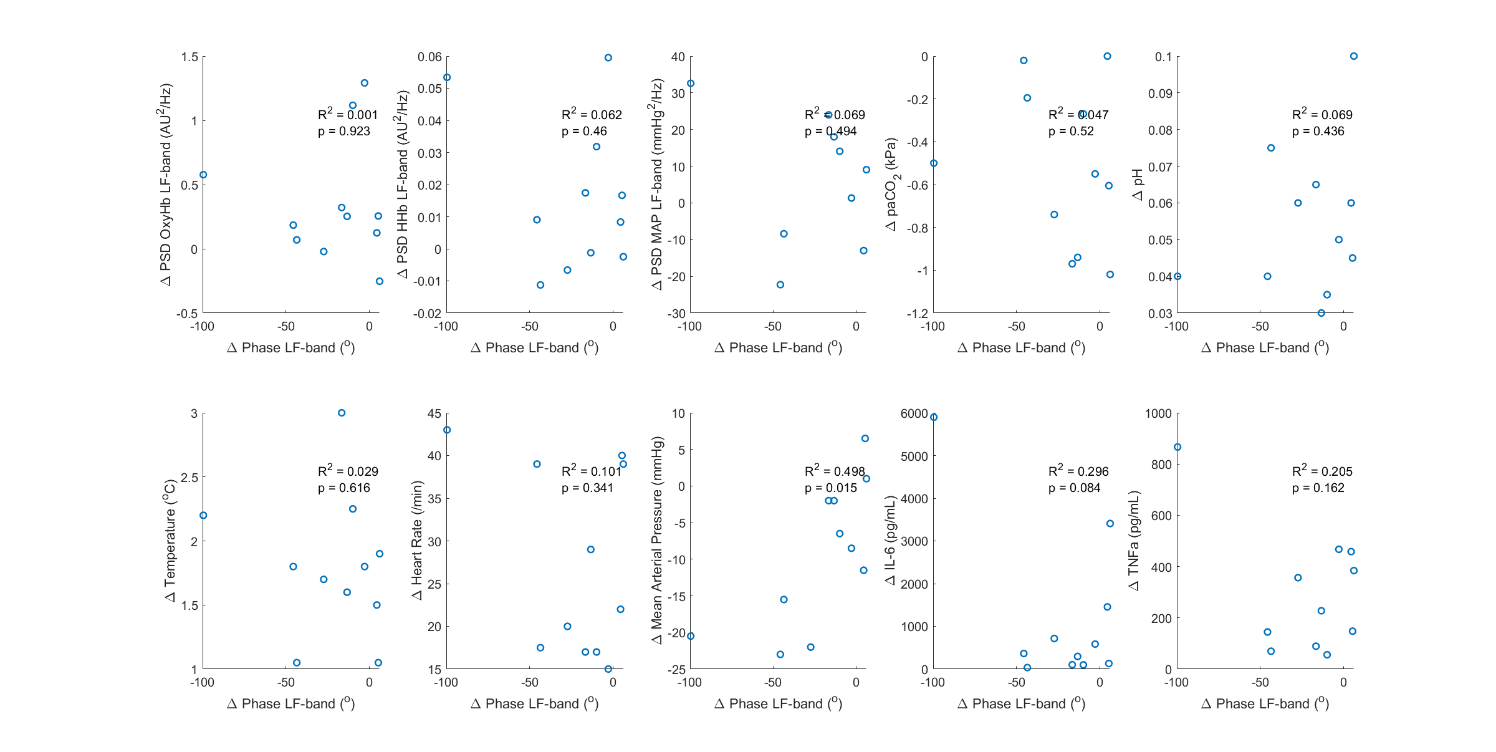
 Supplementary material - Univariate relation between LF-phase and TSI with potential confounders

**Figure S1:** Scatterplots of the change between Time Points 1 (Baseline) and 2 (Systemic inflammation) in phase difference in the LF band and change in several potential hemodynamic confounders:
(A) PSD of OxyHb in the LF-band
(B) PSD of HHb in the LF-band
(C) PSD of MAP in the LF-band
(D) PaCO_2_
(E) pH
(F) Body temperature
(G) Heart rate
(H) MAP
(I) IL-6
(J) TNFα
IL: Interleukin; LF: Low-frequency; VLF: Very low-frequency; PSD: Power Spectral Density; OxyHb: Oxygenated Haemoglobin, HHb: Deoxygenated Haemoglobin; MAP: Mean arterial pressure; PaCO_2_: partial pressure of carbon dioxide in arterial blood; TNFα: Tumour necrosis factor alpha


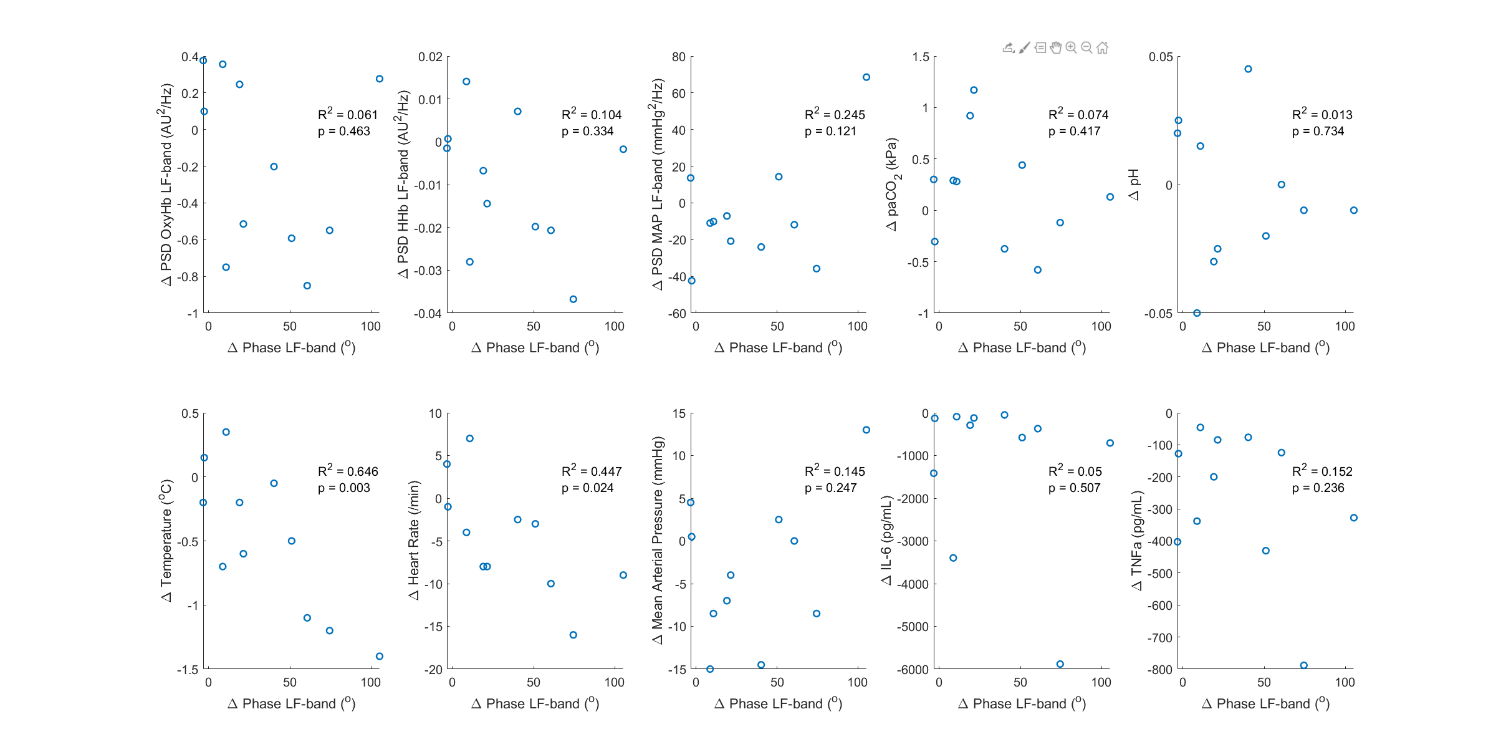


**Figure S2**: Scatterplots of the change between Time Points 2 (Systemic inflammation) and 3 (End-of-experiment) in phase difference in the LF band and change in several potential hemodynamic confounders:
(A) PSD of OxyHb in the LF-band
(B) PSD of HHb in the LF-band
(C) PSD of MAP in the LF-band
(D) PaCO_2_
(E) pH
(F) Body temperature
(G) Heart rate
(H) MAP
(I) IL-6
(J) TNFα
IL: Interleukin; LF: Low-frequency; VLF: Very low-frequency; PSD: Power Spectral Density; OxyHb: Oxygenated Haemoglobin, HHb: Deoxygenated Haemoglobin; MAP: Mean arterial pressure; PaCO_2_: partial pressure of carbon dioxide in arterial blood; TNFα: Tumour necrosis factor alpha


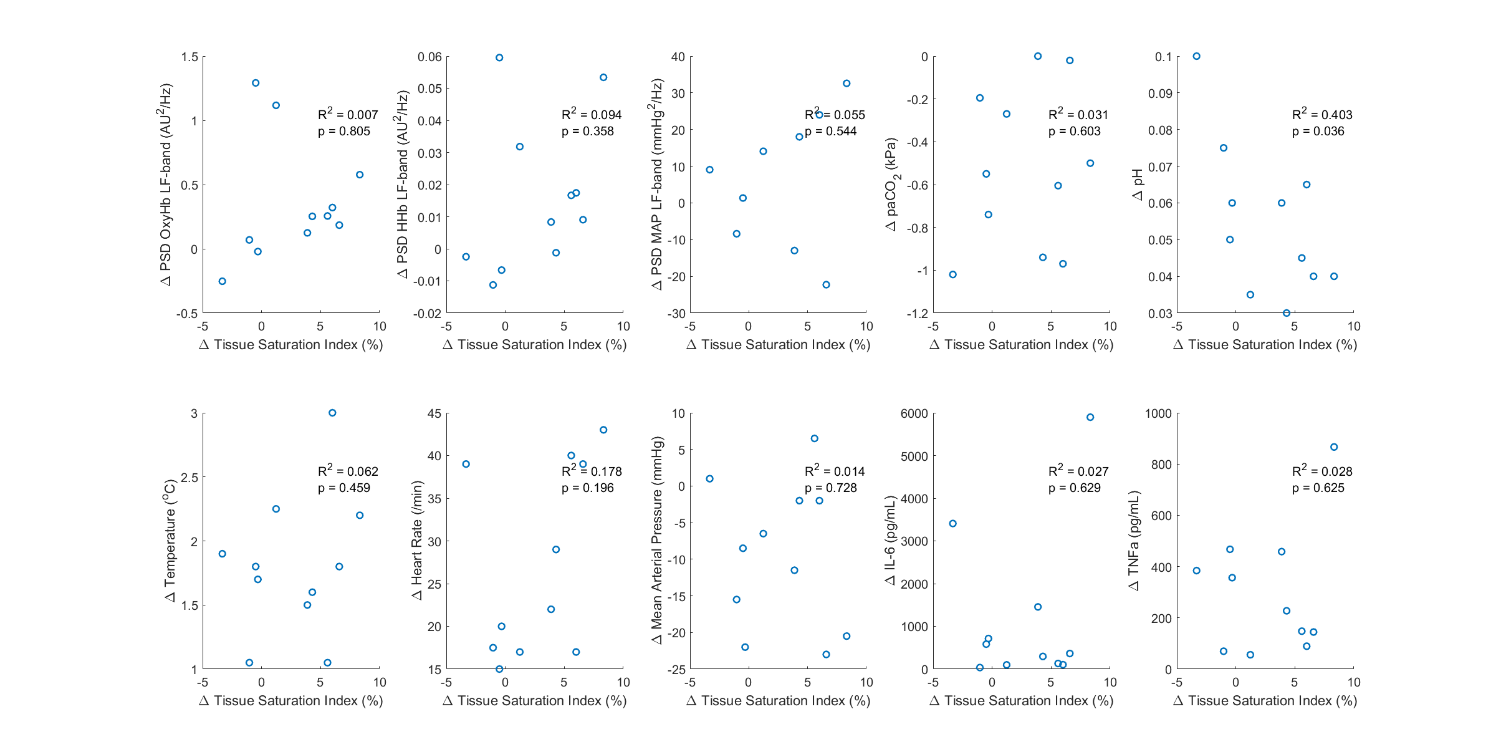


**Figure S3:** Scatterplots of the change between Time Points 1 (Baseline) and 2 (Systemic inflammation) in TSI and change in several potential hemodynamic confounders:
(A) PSD of OxyHb in the LF-band
(B) PSD of HHb in the LF-band
(C) PSD of MAP in the LF-band
(D) PaCO_2_
(E) pH
(F) Body temperature
(G) Heart rate
(H) MAP
(I) IL-6
(J) TNFα
IL: Interleukin; LF: Low-frequency; VLF: Very low-frequency; PSD: Power Spectral Density; OxyHb: Oxygenated Haemoglobin, HHb: Deoxygenated Haemoglobin; MAP: Mean arterial pressure; PaCO_2_: partial pressure of carbon dioxide in arterial blood; TNFα: Tumour necrosis factor alpha


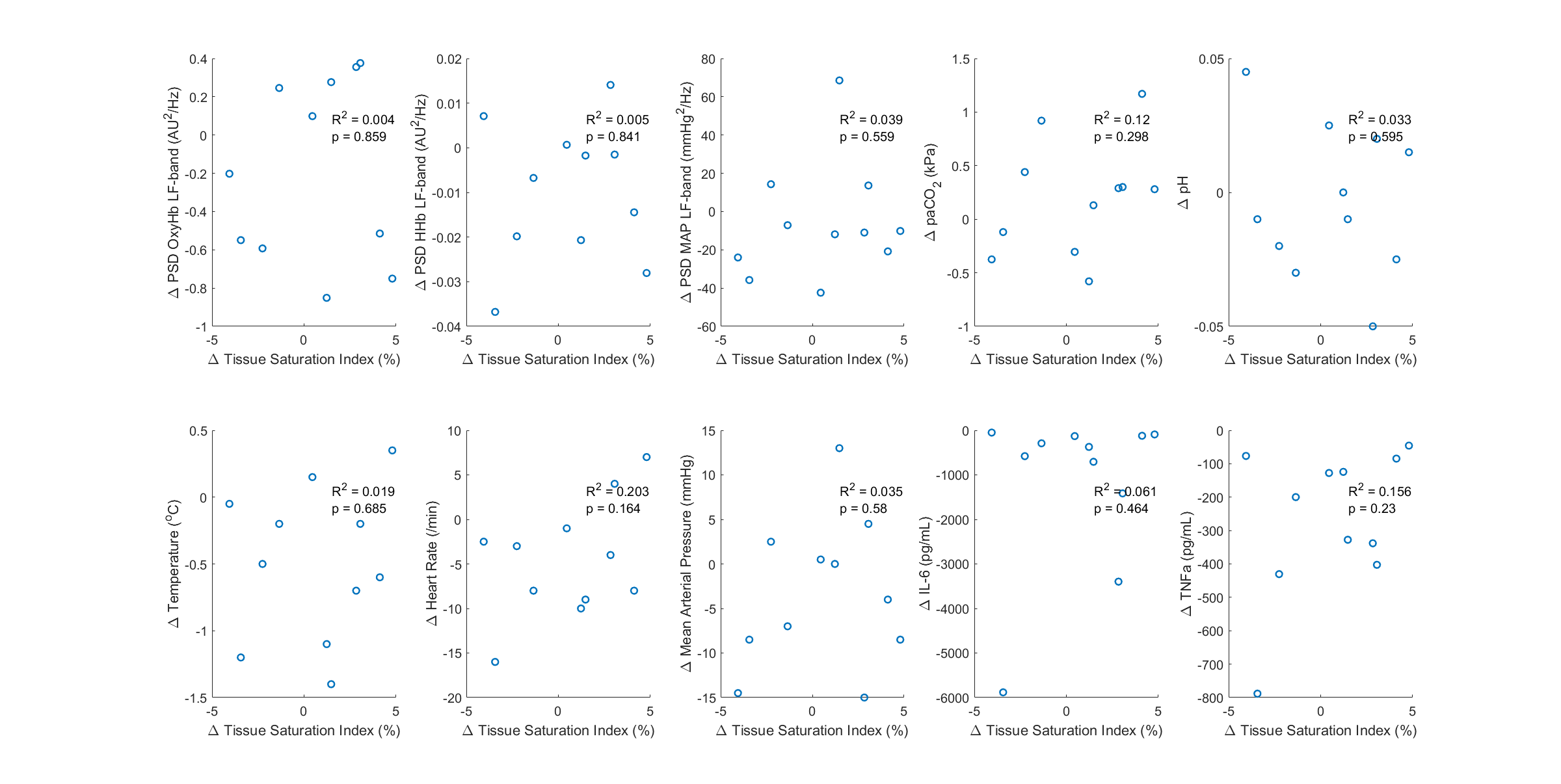


**Figure S4**: Scatterplots of the change between Time Points 2 (Systemic inflammation) and 3 (End-of-experiment) in TSI and change in several potential hemodynamic confounders:
(A) PSD of OxyHb in the LF-band
(B) PSD of HHb in the LF-band
(C) PSD of MAP in the LF-band
(D) PaCO2
(E) pH
(F) Body temperature
(G) Heart rate
(H) MAP
(I) IL-6
(J) TNFα
IL: Interleukin; LF: Low-frequency; VLF: Very low-frequency; PSD: Power Spectral Density; OxyHb: Oxygenated Haemoglobin, HHb: Deoxygenated Haemoglobin; MAP: Mean arterial pressure; PaCO_2_: partial pressure of carbon dioxide in arterial blood; TNFα: Tumour necrosis factor alpha
