# Supplementary material for: Near-Infrared Spectroscopy-Derived Dynamic Cerebral Autoregulation in Experimental Human Endotoxemia—An Exploratory Study
Source: Front Neurol. 2021 Sep 10;12:695705. doi: 10.3389/fneur.2021.695705 (PMC8461327; doi:10.3389/fneur.2021.695705)
Supplement: Supplementary file 2 [file Table_1.docx]

|  |  | Time point 1 Baseline | Time point 2 Systemic inflammation | Time point 3 End-of-experiment |
| --- | --- | --- | --- | --- |
| Coherence VLF | Median (IQR) | 0.23 (0.17 – 0.48) | 0.31 (0.21 – 0.50) | 0.43 (0.23 – 0.47) |
| Coherence LF | Median (IQR) | 0.40 (0.17 – 0.55) | 0.38 (0.16 – 0.62) | 0.34 (0.24 – 0.46) |
| Coherence HF | Median (IQR) | 0.42 (0.33 – 0.51) | 0.46 (0.37 – 0.65) | 0.52 (0.31 – 0.65) |
| Gain VLF | Median (IQR) | 0.13 (0.10 – 0.18) | 0.15 (0.11 – 0.21) | 0.16 (0.11 – 0.18) |
| Gain LF | Median (IQR) | 0.13 (0.11 – 0.17) | 0.16 (0.12 – 0.17) | 0.15 (0.11 – 0.17) |
| Gain HF | Median (IQR) | 0.19 (0.18 – 0.20) | 0.21 (0.16 – 0.24) | 0.20 (0.16 – 0.29) |
| Phase difference VLF | Median (IQR) | 46.8 (21.4 – 91.2) | 45.2 (12.7 – 66.0) | 63.0 (20.7 – 138.3) |
| Phase difference LF | Median (IQR) | 16.2 (3.0 – 52.6) | 3.9 (2.0 – 8.8) | 27.6 (12.7 – 67.5) |
| Phase difference HF | Median (IQR) | 0.01 (0.00 – 1.75) | 0.19 (0.00 – 2.53) | 0.00 (-0.02 – 1.10) |

Supplementary material – NIRS-CA coherence, gain, and phase difference values per frequency band

Table S1: Coherence, gain, and phase difference between oxygenated (OxyHb) and deoxygenated (HHb) haemoglobin of the n=11 participants for the very low (VLF), low (LF), and high (HF) frequency bands for each time point.
